# CT-detected inguinal and femoral hernias in women: prevalence and interobserver agreement

**DOI:** 10.3389/fradi.2026.1957651

**Published:** 2026-10-13

**Authors:** Sara Gómez Milanés, Oreanna Quintero Rojas, Jaime Martínez, Fernando Ortiz, Andrés Felipe González Serna

**Affiliations:** 1 Programa de Radiología e Imágenes Diagnósticas, Fundación Universitaria Sanitas, Bogotá, Colombia; 2 Departamento de Radiología e Imágenes Diagnósticas, Colsanitas, Bogotá D.C., Colombia

**Keywords:** Colombia, diagnostic imaging, femoral hernia, inguinal hernia, observational studies as topic, observer variation, women's health, X-ray computed tomography

## Abstract

**Background:**

Inguinal and femoral hernias in women show a reported diagnostic gap, with systematic evidence supported by cohorts composed of approximately 84% men.

**Materials and methods:**

We conducted a descriptive, retrospective, cross-sectional observational study at a reference center in Bogotá during the 2024–2025 period. We included 494 women, 18 years or older, who had undergone abdominal computed tomography. Double reading by residents was applied, with 2-of-3 majority adjudication between the resident and two independent expert reviewers. Statistical analysis included 95% Wilson confidence intervals, Cohen's kappa with Landis–Koch interpretation, and sensitivity, specificity, positive predictive value, and negative predictive value, reported in accordance with STROBE guidelines.

**Results:**

Resident-diagnosed prevalence was 88 of 494 (17.8%; 95% CI 14.7–21.4). Adjudicated prevalence was 83 of 494 (16.8%; 95% CI 13.8–20.4). Five cases (1.0%) were reclassified as extraperitoneal fat by expert consensus. Findings included indirect inguinal predominance, bilateral distribution in 26.1%, a high proportion of occult hernias, and underreporting in the original radiology report. Resident sensitivity was 100% against both expert reviewers. Specificity was 84.4% against Reviewer 1 and 47.4% against Reviewer 2. Cohen’s kappa for resident vs. Reviewer 1 was 0.878; resident vs. Reviewer 2 was 0.326; and Reviewer 1 vs. Reviewer 2 was 0.408.

**Conclusions:**

The radiological prevalence in women sits above the classical clinical range. Extraperitoneal fat is an overlooked differential diagnosis in female cohorts and accounts for a large part of interreviewer discordance. The 2-of-3 rule works as a replicable method for diagnostic studies with limited resources.

## Introduction

Inguinal and femoral hernias are among the most frequent surgical pathologies worldwide, with prevalence variations across geographical regions (1). International HerniaSurge guidelines frame them as a global clinical problem requiring an organized response (2, 3). Despite aggregate figures, prevalence differs markedly by sex, age, comorbidities, and socioeconomic context (4). Latin American epidemiological evidence remains scarce, and sex-disaggregated data are even rarer.

Epidemiological literature on inguinal hernia has historically been built on predominantly male populations, with lifetime prevalence of 27%–43% in men vs. 3%–6% in women (4, 5). A recent reanalysis confirmed that women have femoral hernia at a substantially higher rate than men (5). The female inguinal canal has distinct anatomy: The round ligament of the uterus replaces the spermatic cord, accompanied by the round ligament artery, branches of the ilioinguinal nerve, and the embryological canal of Nuck (6). These variants require closer radiologic scrutiny in women than in men (7).

Physical examination performs poorly in women, with occult presentations often leading to attribution of inguinal or pelvic pain to gynecological, musculoskeletal, or functional causes for months or years (8, 9). When clinical diagnosis is not evident, imaging becomes cardinal to identify occult hernias, differentiate inguinal from femoral, or detect complications (10, 11). Computed tomography (CT) offers the greatest opportunity for incidental hernia diagnosis, with reported sensitivity of 80% and specificity of 65% (11), and is frequently performed for non-hernia indications.

The systematic review by Piga et al. reported CT sensitivity of 48%–98% and specificity of 25%–100%, but 84% of the pooled population was men (12). Evidence on CT performance specifically in female populations is limited, precisely where the diagnosis may be more complex. Two entities merit specific attention on CT of the female inguinal region: occult hernias, associated with chronic pain and delayed care (8, 13), and extraperitoneal fat protrusion in the inguinal canal, which lacks a true peritoneal sac but is often misclassified as hernia (14, 15).

We aimed to estimate the CT-based prevalence of inguinal and femoral hernias in adult women, describe their anatomical and tomographic characteristics in the subset with complete characterization, and assess interobserver agreement between the initial resident readings and two independent expert reviewers.

## Materials and methods

This was an observational, descriptive, retrospective, cross-sectional study, reported in accordance with STROBE guidelines for cross-sectional studies (16), with a narrative framework prepared according to SANRA criteria (17). The cohort was drawn from abdominal CT scans performed for heterogeneous clinical indications at a reference center covering the Bogotá–Sabana axis. The image acquisition window was January 2024 through December 2025. Data collection and analysis were carried out between 2024 and 2026.

Inclusion criteria were female sex, age ≥18 years, and availability of simple or contrast-enhanced CT of total abdomen or pelvis. The exclusion criterion was inadequate technical quality (incorrect segmentation of structures of interest, geometric inaccuracy, or positional inconsistencies from patient displacement). Of 500 tomographic studies identified in the temporal window, 6 (1.2%) were excluded and 494 (98.8%) were included. The realized sample size exceeded the *a priori* target of 184 patients (computed for a proportion with Z = 1.96, *P* = 0.139, margin of error d = 0.05), thereby increasing statistical power without modifying the original objectives.

Data sources included the institutional PACS for imaging and the electronic clinical record for clinical data. Information handling complied with Colombian Resolution 1995 of 1999 and Statutory Law 1581 of 2012 on personal data protection. The presence and type of groin hernia were determined by tracing the protruding structures on contiguous CT images and identifying the anatomic neck. A CT-positive hernia was defined as focal protrusion of a peritoneal sac, with or without fat or visceral content, through a recognized inguinal or femoral defect. An indirect inguinal hernia arose lateral to the inferior epigastric vessels through the deep inguinal ring; a direct inguinal hernia arose medial to these vessels through Hesselbach's triangle. Inguinal hernias were located anterior to the inguinal ligament, whereas femoral hernias passed posterior/inferior to the ligament through the femoral canal, medial to the common femoral vein (18, 19). Fat extending into the inguinal canal without a demonstrable peritoneal sac or hernia neck was classified as extraperitoneal fat protrusion rather than a true hernia (14, 15) (Figure 1).

**Figure 1:**
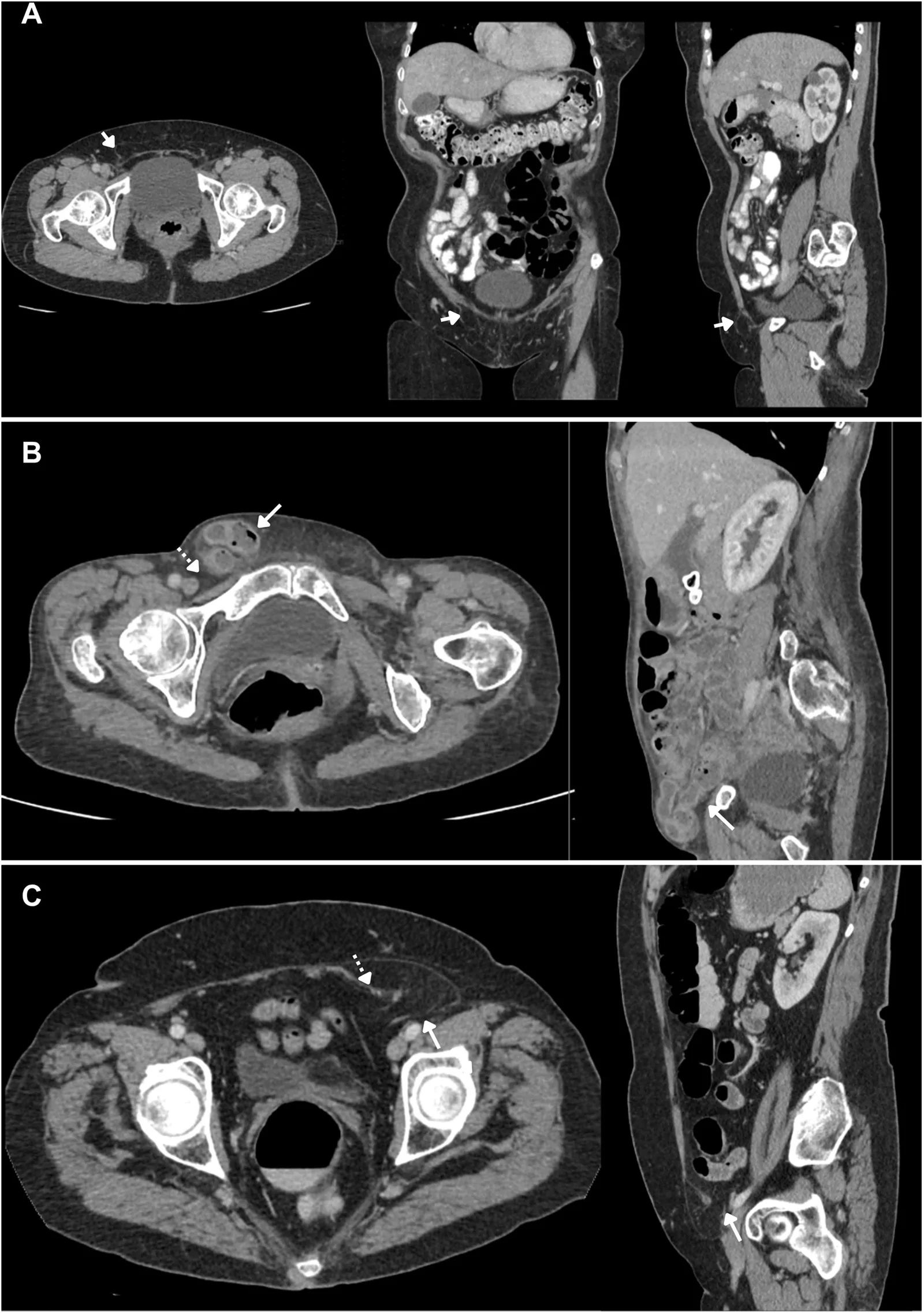
Radiological distinction between extraperitoneal fat protrusion and true inguinal hernias. **(A)** Computed tomography (CT) images demonstrating extraperitoneal fat protrusion. Solid arrows indicate fat extending into the inguinal canal, which lacks a true hernia sac or intraperitoneal content. **(B)** CT images of a direct inguinal hernia. Solid arrows outline the distinct hernia sac and wall defect, located medial to the inferior epigastric vessels (dashed arrow). **(C)** CT images of an indirect inguinal hernia. Solid arrows indicate the true hernia sac protruding lateral to the inferior epigastric vessels (dashed arrow).

The assessment incorporated three reading sources: (i) the original radiologic report issued during clinical care, (ii) independent blinded rereading by two residents in training, and (iii) subsequent expert review and adjudication. An occult hernia was defined operationally as a CT-positive hernia for which neither a preexisting diagnosis nor clinical suspicion of groin hernia was documented before CT; whether the finding was mentioned in the original radiology report was recorded as a separate variable. Clinical and surgical data were reviewed when available, but operative confirmation was not required for inclusion and was not systematically available. Therefore, not all CT-positive hernias were surgically confirmed. The final study classification was imaging-based and derived from the adjudication process described in the following; sensitivity, specificity, positive predictive value (PPV), and negative predictive value (NPV) represent performance relative to expert or adjudicated imaging classifications, not accuracy against an operative gold standard.

The same CT interpretation criteria were applied in the resident and expert rereadings. The denominator for the cohort-level CT frequency estimate was the 494 included CT studies. Descriptive analyses of age, laterality, type and subtype, sac content, complications, occult status, and documentation in the original radiology report were restricted to the 69 cases with complete characterization. Pairwise reader performance analyses included only studies with both readings available (*n* = 96 for Reviewer 1 and *n* = 78 for Reviewer 2); the three-reader combination analysis used the 78 studies with all three readings. Missing characterization fields and unavailable reader assessments were not imputed.

A protocol amendment introduced expert adjudication. During validation, a high rate of discordance was observed between residents and the first expert reader (Reviewer 1), with a systematic reclassification pattern toward extraperitoneal fat. A second independent expert (Reviewer 2) was incorporated to evaluate the same cases blindly, plus a random sample of resident-Normal cases. The adopted rule was 2-of-3 majority: resident, Reviewer 1, and Reviewer 2. When only one expert reading was available, the verdict followed that single expert reading and the case was flagged as pending adjudication by the missing reviewer. Operationalization of variables is detailed in Table 1.

**Table 1:** Operationalization of variables included in the analysis.

Group	Variable	Level of measurement	Nature	Scale	Categories or unit	Source
Identification	Age	Quantitative	Discrete	Continuous	Completed years	Protocol
Hernia anatomy	Hernia type	Qualitative	Nominal	Not applicable	1 = Inguinal, 2 = Femoral, 3 = Inguinal plus femoral	Protocol
Hernia anatomy	Inguinal subtype	Qualitative	Nominal	Not applicable	1 = Direct, 2 = Indirect, 3 = Mixed, Not applicable for femoral	Protocol
Hernia anatomy	Laterality	Qualitative	Nominal	Not applicable	1 = Right, 2 = Left, 3 = Bilateral	Amendment
Hernia anatomy	Defect size	Quantitative	Continuous	Continuous	Millimeters	Amendment
CT findings	Hernia sac content	Qualitative	Nominal	Not applicable	1 = Fat only, 2 = Bowel content, 3 = Mixed, 4 = Other	Protocol
CT findings	Inflammatory changes	Qualitative	Nominal	Not applicable	1 = Yes, 2 = No	Protocol
CT findings	Adjacent free fluid	Qualitative	Nominal	Not applicable	1 = Yes, 2 = No	Protocol
Complications	Complication present	Qualitative	Nominal	Not applicable	1 = Yes, 2 = No	Protocol
Complications	Complication type	Qualitative	Nominal	Not applicable	1 = Incarceration, 2 = Strangulation, 3 = Bowel obstruction, 4 = Omental infarction, 5 = Stercoral fistula, 6 = Solid organ herniation	Protocol
Clinical context	CT indication	Qualitative	Nominal	Not applicable	Free text categorized into seven algorithmic groups: abdominal pain, inguinal or pelvic pain, oncologic control or follow-up, infectious or inflammatory process, constitutional symptoms, trauma, other or not recorded	Amendment
Clinical context	Prior abdominal wall surgery	Qualitative	Nominal	Not applicable	1 = Yes, 2 = No	Protocol
Clinical context	Hernia reported in original report	Qualitative	Nominal	Not applicable	1 = Yes, 2 = No	Amendment
Clinical context	Occult hernia	Qualitative	Nominal	Not applicable	1 = Occult (not clinically suspected), 2 = Not occult (symptomatic or known)	Protocol

Protocol = variable prespecified in the protocol approved by the Ethics Committee; Amendment = variable incorporated during execution. CT: computed tomography.

Statistical analysis was performed in R 4.5.1. All proportions include 95% confidence intervals by the Wilson method (20), implemented with DescTools::BinomCI. Interobserver concordance was estimated with Cohen's kappa (21), implemented with psych::cohen.kappa, interpreted using Landis and Koch ranges (poor <0.20; fair 0.20–0.39; moderate 0.40–0.59; substantial 0.60–0.79; almost perfect ≥0.80) (22). Sensitivity, specificity, positive predictive value, and negative predictive value of the resident against the adjudicated verdict were computed with 95% Wilson CIs. This study was classified as no-risk research under Colombian Resolution 8430 of 1993 and was approved by the Institutional Research Ethics Committee of Fundación Universitaria Sanitas.

## Results

Of 500 tomographic studies identified in the 2024–2025 window, 6 (1.2%) were excluded for inadequate technical quality, leaving 494 (98.8%) for analysis. Figure 2 presents the flow diagram from initial identification through 2-of-3 adjudication, including reclassification as extraperitoneal fat.

**Figure 2:**
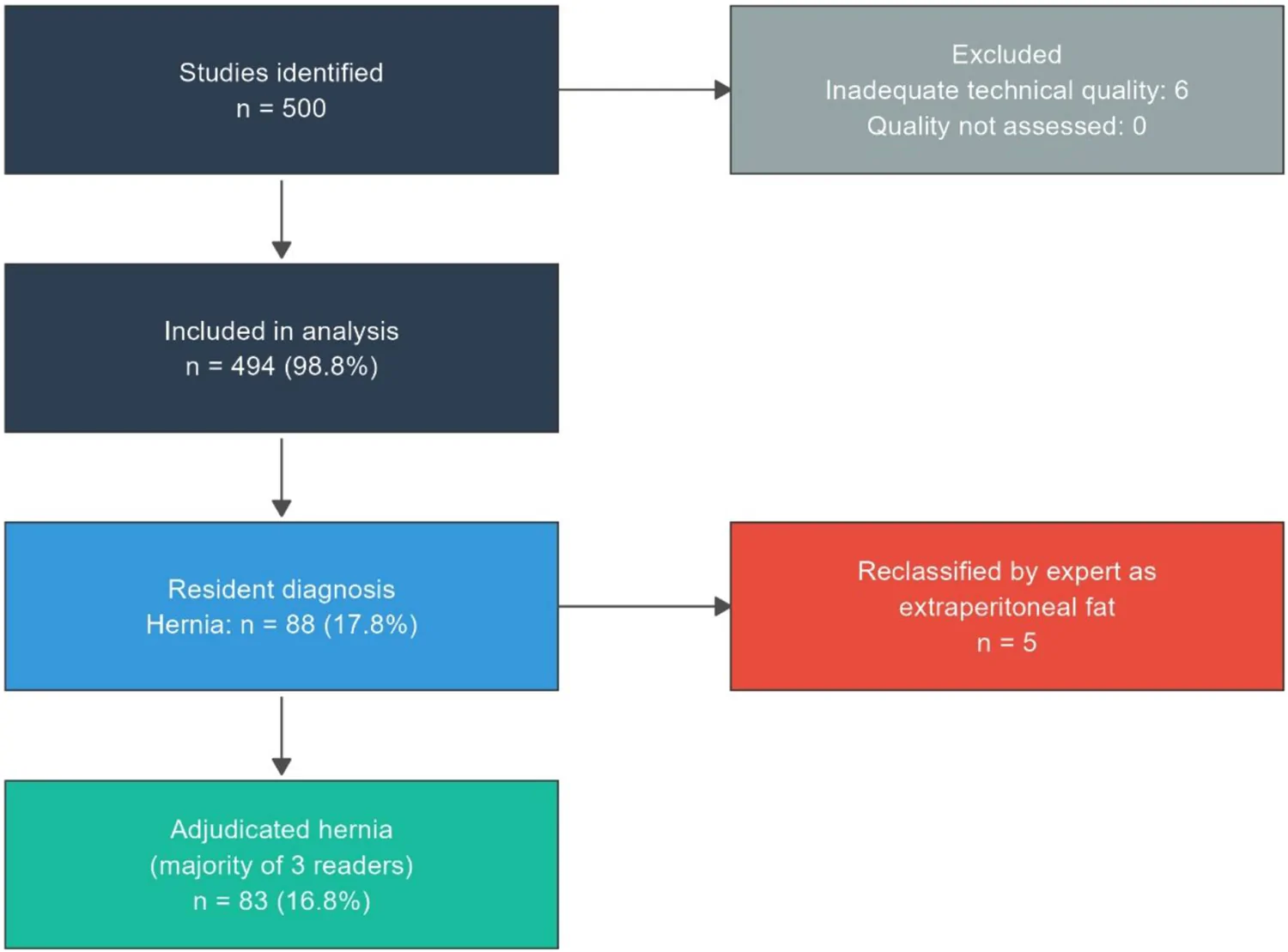
Flow diagram of inclusion, adjudication, and reclassification as extraperitoneal fat. Identified studies and their classification following resident reading, expert reading, and application of the 2-of-3 adjudication rule.

Hernia prevalence diagnosed by the resident reader was 88/494 (17.8%; 95% Wilson CI 14.7–21.4). After 2-of-3 adjudication, prevalence was adjusted to 83/494 (16.8%; 95% CI 13.8–20.4). Reclassification as extraperitoneal fat reached 5/494 (1.0%; 95% CI 0.4–2.3): All five cases had been labeled as hernia by the resident and consistently reclassified as Normal by both expert reviewers. Eighteen resident-hernia cases remained pending Reviewer 2 rereading; in accordance with the prespecified rule, their verdict followed the available Reviewer 1 reading. Resident-hernia cases without any expert reading retained the resident's classification.

Operative confirmation was not systematically available and was not used as the reference standard. Consequently, adjudicated cases should be interpreted as CT-positive diagnoses after imaging review rather than surgically confirmed hernias.

Unless otherwise specified, all anatomical, clinical, and CT-characteristic percentages reported for this subgroup use *n* = 69 as the denominator rather than the full cohort of 494. Reader performance and agreement analyses use the available-case denominators specified in Tables 2, 3.

**Table 2:** Reader performance metrics within the pairwise validation subsets.

Comparison	*n*	Sensitivity	Specificity	PPV	NPV	Cohen's kappa (95% CI)	Interpretation
Resident vs. Reviewer 1	96	64/64 = 100.0% (95% CI 94.3–100.0)	27/32 = 84.4% (95% CI 68.2–93.1)	64/69 = 92.8% (95% CI 84.1–96.9)	27/27 = 100.0% (95% CI 87.5–100.0)	0.878 (0.775–0.981)	Almost perfect (Landis–Koch)
Resident vs. Reviewer 2	78	21/21 = 100.0% (95% CI 84.5–100.0)	27/57 = 47.4% (95% CI 35.0–60.1)	21/51 = 41.2% (95% CI 28.8–54.8)	27/27 = 100.0% (95% CI 87.5–100.0)	0.326 (0.187–0.466)	Fair (Landis–Koch)
Resident vs. adjudicated verdict	96	64/64 = 100.0% (95% CI 94.3–100.0)	27/32 = 84.4% (95% CI 68.2–93.1)	64/69 = 92.8% (95% CI 84.1–96.9)	27/27 = 100.0% (95% CI 87.5–100.0)	0.878 (0.775–0.981)	Almost perfect (Landis–Koch)
Reviewer 1 vs. Reviewer 2 (inter-expert)	78	—	—	—	—	0.408 (0.254–0.562)	Moderate (Landis–Koch)

95% Wilson CI. Kappa according to Cohen; interpretation according to Landis–Koch (22). Denominators reflect available paired readings: *n* = 96 for Reviewer 1 and the available adjudicated verdict, and *n* = 78 for Reviewer 2 and the inter-expert comparison. Missing reader assessments were not imputed.

**Table 3:** Combinations of the three readings and majority verdict (2 of 3) in the complete three-reader subset (*n* = 78).

Resident	*n*	Adjudicated verdict (2 of 3)
Normal	27	Normal
Hernia	25	Hernia
Hernia	21	Hernia
Hernia	5	Normal

Only cases with all three readings available.

The CT-positive cohort with complete characterization comprised 69 women. Age was not normally distributed (Shapiro–Wilk *p* < 0.05), with mean 70.2 years (SD 14.0) and median 71 years (IQR 62–81), range 36–98. Figure 3 shows the age distribution as a boxplot with individual observations. Distribution concentrated in advanced age, with 60–79 years as the modal group followed by ≥80 years. Abdominal wall surgical antecedent was documented in 6/69 (8.7%; 95% CI 4.0–17.7). The most frequent CT indication was abdominal pain, followed by oncologic follow-up; specific hernia-related indications (inguinal/pelvic pain, hernia suspicion) collectively represented a small proportion, consistent with the non-directed nature of the cohort.

**Figure 3:**
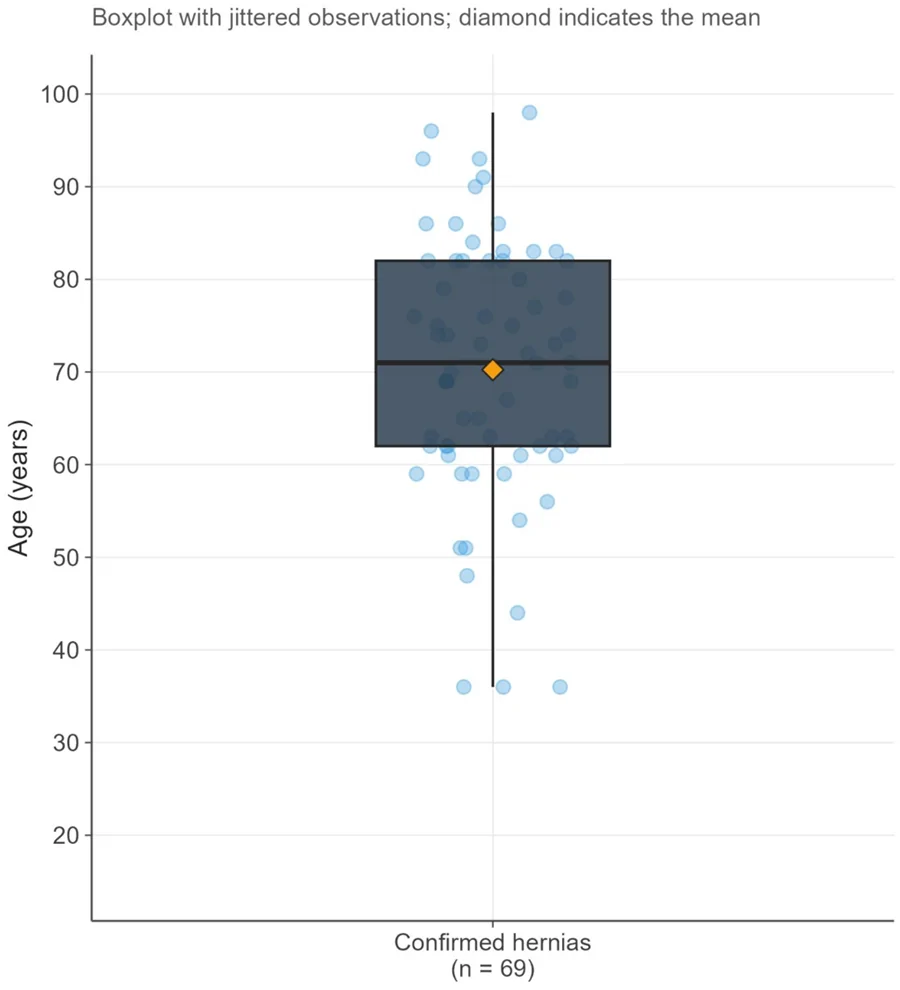
Age distribution in the completely characterized CT-positive subset (*n* = 69). Boxplot with individual observations; the orange diamond indicates the mean.

Type distribution showed inguinal predominance, with a smaller presence of femoral and mixed inguinal-plus-femoral configurations. Indirect inguinal was the most frequent subtype, followed by direct and mixed. Figure 4 details type and subtype distribution. Laterality showed right, left, and bilateral cases; bilaterality reached 26.1% (18/69). Defect size could be quantified numerically in a subset of cases with clean single reports; free-text non-parseable cases were excluded from the quantitative estimate. Tomographic complications were infrequent in the cohort and predominantly consisted of intestinal obstruction, without documented strangulation.

**Figure 4:**
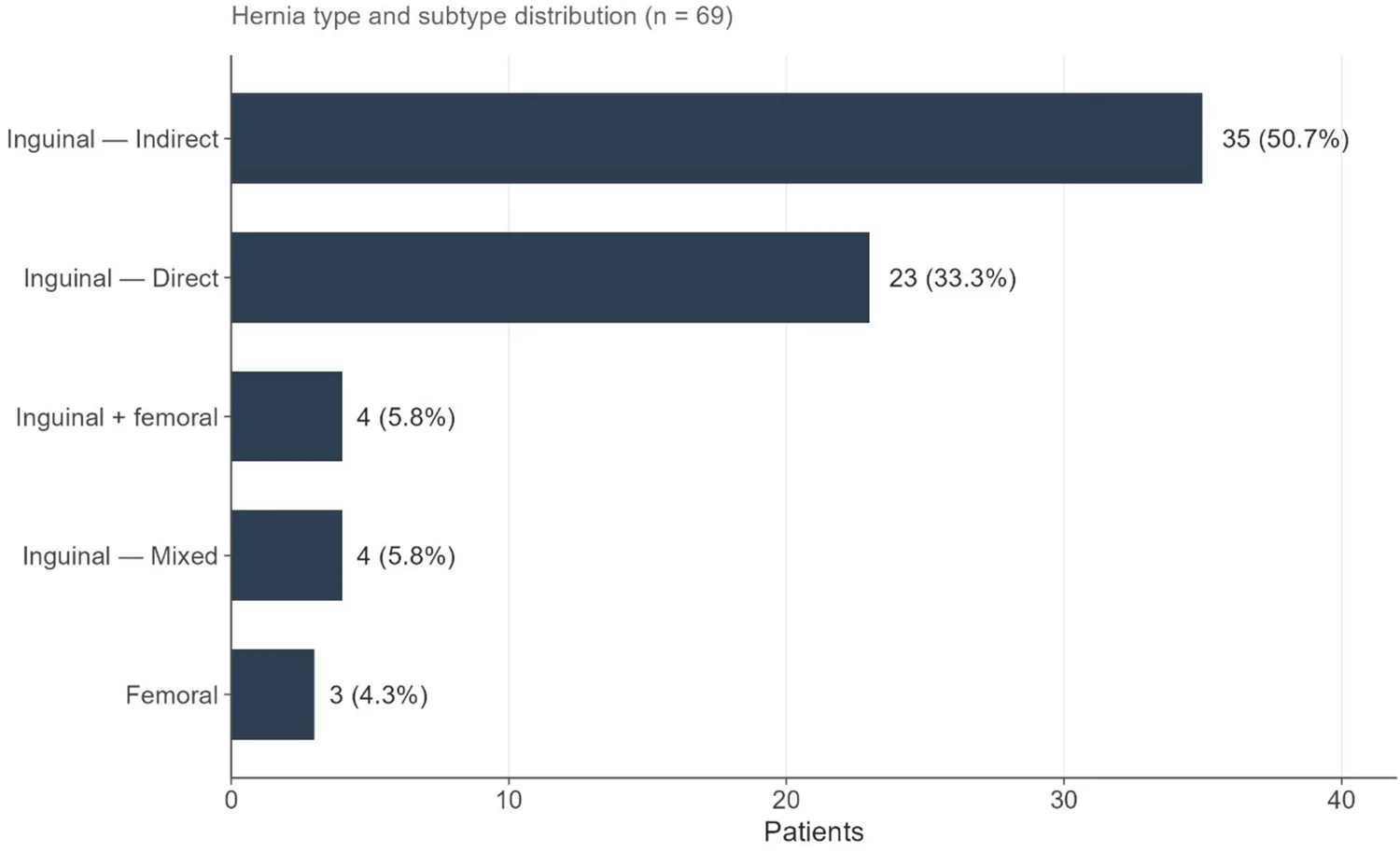
Distribution of hernia types and subtypes in the completely characterized subset (*n* = 69). Combination of type (inguinal, femoral, mixed) and inguinal subtype (direct, indirect, mixed).

Occult status was defined by the absence of a documented pre-CT diagnosis or clinical suspicion of groin hernia, regardless of whether the finding was described in the original radiology report. Figure 5 compares this clinical classification with original report documentation. A substantial proportion of retrospectively detected CT-positive hernias had not been included in the original report, illustrating the incidental reporting problem in routine practice.

**Figure 5:**
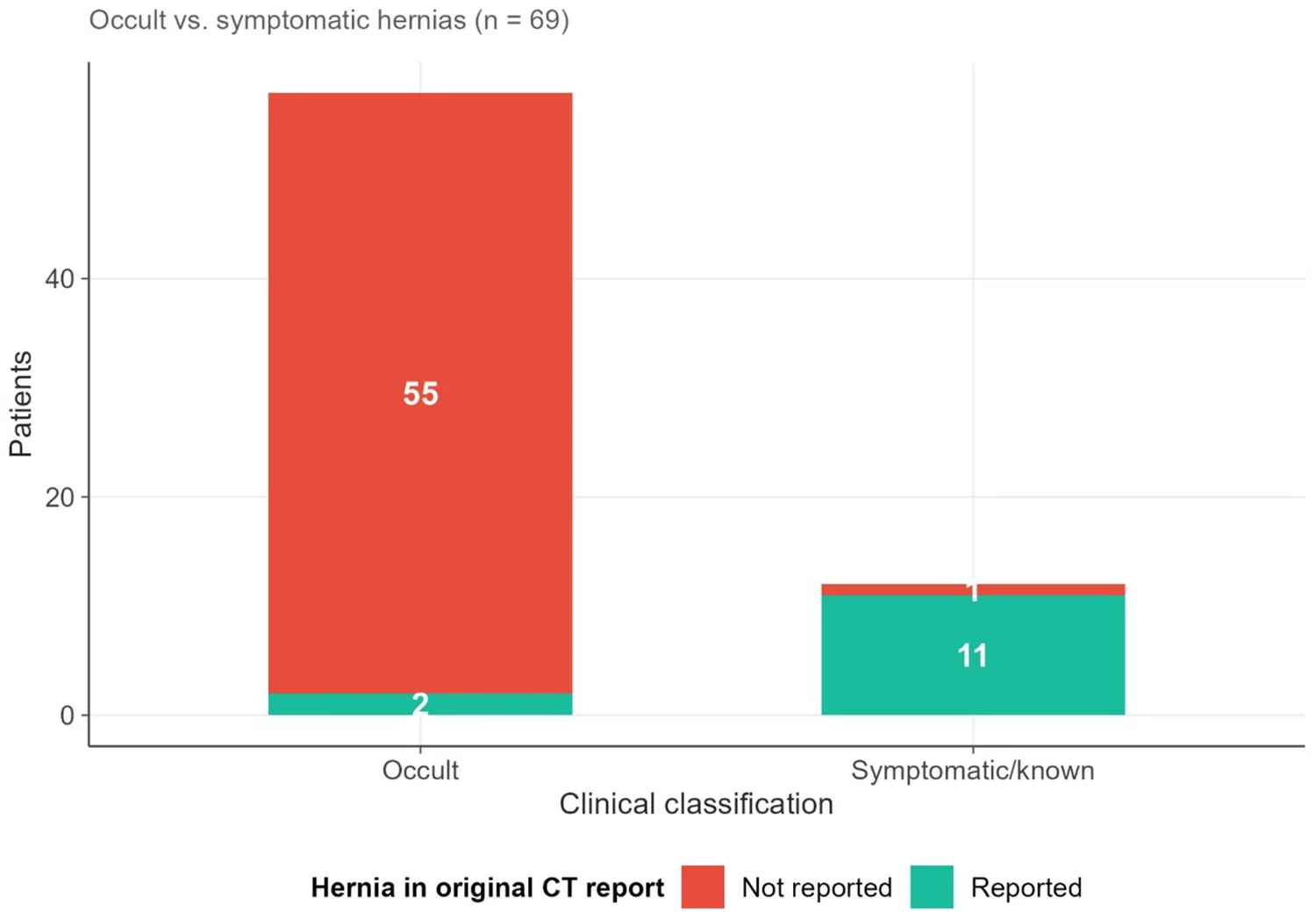
Occult vs. symptomatic hernias in the completely characterized subset (*n* = 69). Comparison between clinical classification and documentation in the original radiology report.

Resident sensitivity vs. Reviewer 1 was 100%, with specificity 84.4% (95% Wilson CI 68.2–93.1), PPV 92.8% (95% CI 84.1–96.9), and NPV 100% (95% CI 87.5–100.0), on 96 cases with dual reading. Resident sensitivity vs. Reviewer 2 remained 100% but specificity dropped to 47.4% (95% CI 35.0–60.1), with PPV 41.2% (95% CI 28.8–54.8) and NPV 100%, on 78 dual-read cases. These differences are consistent with a stricter diagnostic threshold applied by Reviewer 2 and a tendency to reclassify small hernias as extraperitoneal fat. Figure 6 and Table 2 present validity metrics with 95% CIs.

**Figure 6:**
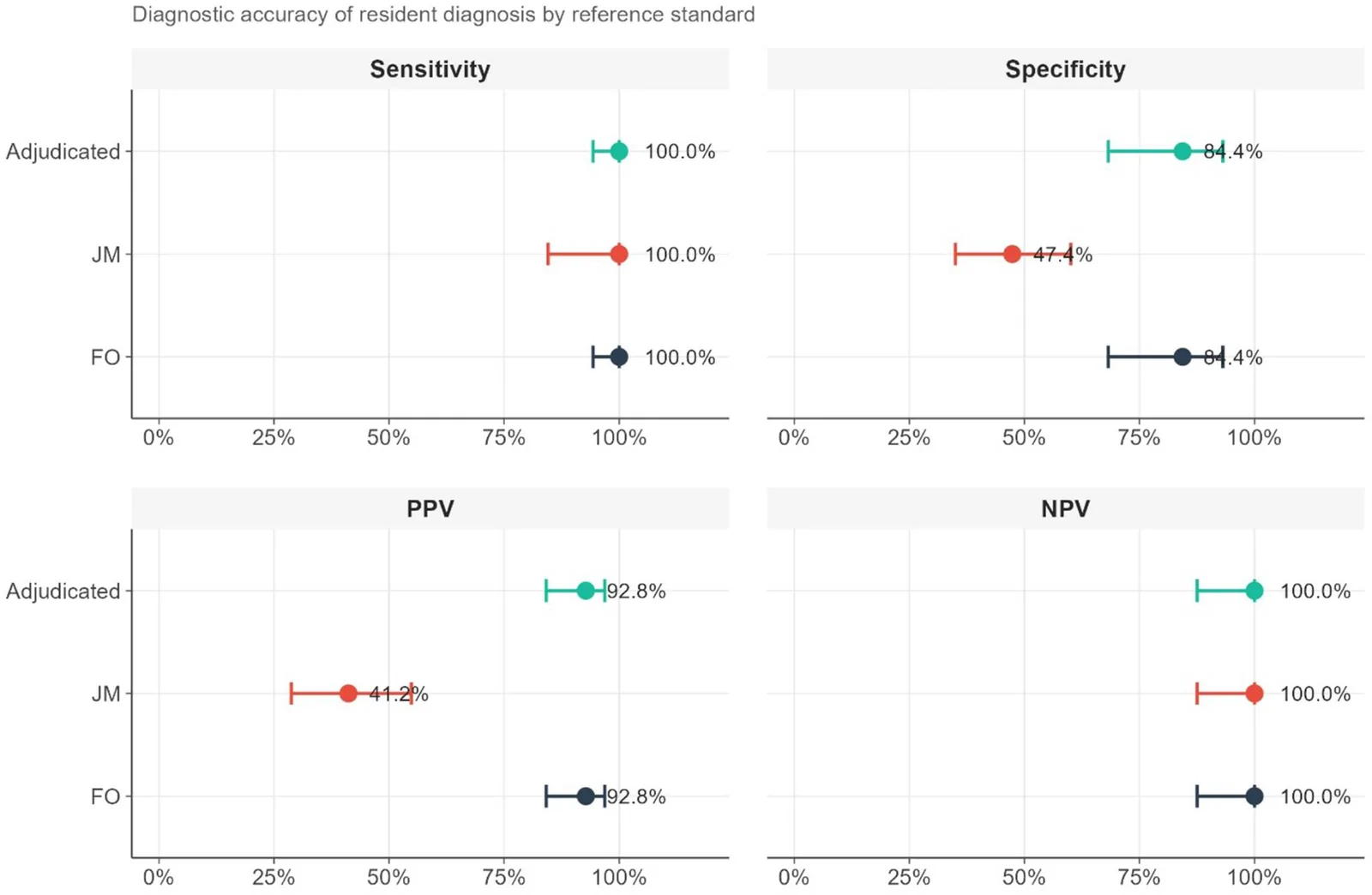
Reader performance metrics by imaging reference classification and available case denominator. Sensitivity, specificity, PPV, and NPV with 95% Wilson CI against Reviewer 1 (*n* = 96), Reviewer 2 (*n* = 78), and the available adjudicated verdict (*n* = 96). FO, Reviewer 1; JM, Reviewer 2.

Interobserver concordance by Cohen's kappa showed contrasting agreement ranges: resident vs. Reviewer 1, kappa 0.878 (95% CI 0.775–0.981), almost perfect according to Landis–Koch; resident vs. Reviewer 2, kappa 0.326 (95% CI 0.187–0.466), fair; and inter-expert Reviewer 1 vs. Reviewer 2, kappa 0.408 (95% CI 0.254–0.562), moderate. Figure 7 and Table 3 detail the combinations of the three readings and the adjudicated verdicts. The most frequent combination was Resident-Hernia + Reviewer 1-Hernia + Reviewer 2-Normal (25 cases, adjudicated verdict Hernia by majority). The Normal-by-majority combination (Resident-Hernia + Reviewer 1-Normal + Reviewer 2-Normal) summed 5 cases, matching the magnitude of extraperitoneal fat reclassification.

**Figure 7:**
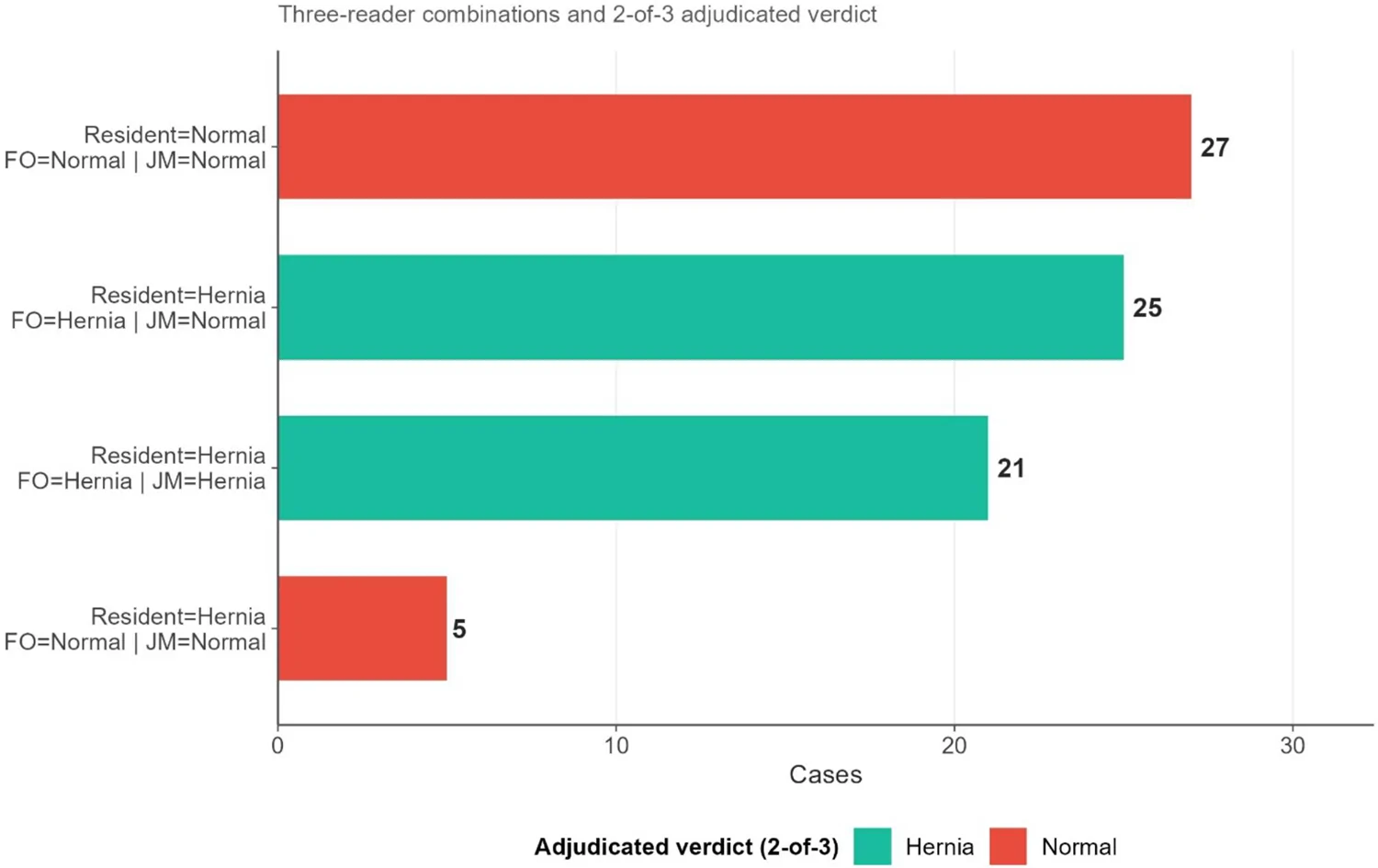
Combinations of the three readings and adjudicated verdict by the 2-of-3 rule in the complete three-reader subset (*n* = 78). Horizontal bars show each reading combination and the final majority verdict. FO, Reviewer 1; JM, Reviewer 2.

## Discussion

This study characterized, for the first time in a Colombian female cohort, the prevalence and distribution of inguinal and femoral hernias identified by abdominal CT at a reference center of the Bogotá–Sabana axis. The prevalence diagnosed by the resident reader was 17.8%; after 2-of-3 adjudication, it was adjusted to 16.8%, with 1.0% reclassification as extraperitoneal fat. Patients were concentrated in advanced age, with predominance of indirect inguinal hernias, a high proportion of occult cases, and notable underreporting in the original radiologic report. Interobserver concordance ranged from almost perfect to fair depending on the expert reviewer.

The adjudicated prevalence of 16.8% widely exceeds the classical clinical range of 3%–6% for inguinal hernia in women (4, 5). This excess reflects the incidental nature of the cohort: By capturing all CT scans performed for heterogeneous indications without filtering by hernia suspicion, the figure estimates radiologic prevalence rather than clinically manifest prevalence. Piga et al. reported CT sensitivity of 48%–98% and specificity of 25%–100% in a pooled population with 84% men (12), leaving limited margin to calibrate expectations specific to women. In our cohort, femoral hernias accounted for 4.3% of CT-positive cases after adjudication, plus 5.8% mixed inguinal-plus-femoral configurations, reproducing the asymmetry documented by the recent sex reanalysis of femoral rates (5).

Median age of 71 years was markedly higher than the median of 52 years reported by Ghafoor and colleagues in a mixed cohort with 28% women (23). Concentration in the 60–79 age group and the substantial ≥80 group is consistent with the bimodal incidence distribution of inguinal hernia in the general literature and with postmenopausal connective tissue and pelvic floor changes that predispose to late manifestation of previously silent defects. Bilaterality of 26.1% is a novel figure for tomographic bilaterality in a female cohort not selected by hernia suspicion, consistent with surgical series in which contralateral exploration reveals occult hernia in non-negligible fractions (24).

The high proportion of occult hernias—defined by the absence of a documented pre-CT diagnosis or clinical suspicion, analyzed separately from omission in the original radiology report—is consistent with prior evidence on variability in radiologic reporting (13) and with explicit calls for early diagnosis and treatment (8). Our figure aligns with Garvey's prospective series, which documented high CT detection of occult hernias in patients with unexplained inguinal pain (25). HerniaSurge guidelines position imaging as part of the diagnostic algorithm when clinical doubt exists (2, 3) but do not provide an explicit standard for incidental reporting; this operational gap, combined with underestimation of hernias in women, partly explains the documented underreporting.

The most specific contribution of this study is the systematic documentation of the extraperitoneal fat reclassification pattern. Five cases labeled as hernia by the resident reader were consistently reclassified as Normal by both expert reviewers, in the context of extraperitoneal fat in the inguinal canal without a true peritoneal sac. This pattern echoes the spermatic cord lipoma literature—developed mainly in male populations—which recognizes extraperitoneal fat protrusion as anatomically distinct from hernia (14, 15). Its female equivalent (peri-round-ligament protrusion, inguinal-canal fat infiltration) has been underrepresented, likely explaining its late reclassification in cohorts like ours. Adopting the diagnostic pair “true inguinal hernia vs. extraperitoneal fat protrusion” in routine reporting could reduce prevalence overestimation and improve surgical pathway efficiency, especially in female cohorts.

Interobserver concordance range warrants detailed interpretation. With the same resident as reference, the gradation indicates that variability does not depend primarily on the primary reader's training but on the diagnostic threshold applied by each expert. Reviewer 2 maintained a more conservative criterion penalizing overestimation by extraperitoneal fat, while Reviewer 1 approached the resident's diagnostic sensitivity. This is coherent with prior literature: Ghafoor and colleagues reported a gap of nearly 18 percentage points in sensitivity between three radiologists applying identical criteria (23). Our study formally quantifies the variability with Cohen's kappa (21) and 95% Wilson CIs (20) in a female cohort. The variability is not random: It concentrates on a specific anatomic pattern (true hernia vs. extraperitoneal fat) that can be characterized and potentially reduced through structured training. Table 4 synthesizes the quantitative dialogue between our findings and the reference literature.

**Table 4:** Quantitative differences between the present study's findings and the reference literature.

Compared aspect	Present study	Reference literature	Direction of comparison
Radiological hernia prevalence in women	16.8% adjudicated (95% CI 13.8–20.4) in 494 included studies	3%–6% classical clinical range; 92% PPV for CT in clinically undiagnosed inguinal pain (Garvey)	Consistent with the incidental nature of the cohort; expands the radiological evidence in women
Distribution by anatomical type	89.9% inguinal, 4.3% femoral, 5.8% inguinal plus femoral	Femoral hernia proportionally more frequent in women than men (sex-specific reanalysis)	Consistent with the reported asymmetry; provides a specific figure in an unselected female cohort
Proportion of occult hernias	Majority fraction of the cohort, with substantial underreporting in the original radiology report	Variability and underestimation in radiological reporting of occult hernias described in prior literature	Expands the prior finding by quantifying the magnitude in a Colombian female cohort
Resident sensitivity vs. expert	100% vs. Reviewer 1 and vs. Reviewer 2	Range 48%–98% in the Piga systematic review; 68%–86% in Ghafoor depending on reader	At the upper end of the prior range; explainable by the partial verification design
Specificity vs. Reviewer 2	47.4% (95% CI 35.0–60.1)	Range 25%–100% in the Piga systematic review	At the lower end of the range, due to Reviewer 2's more conservative criterion for extraperitoneal fat
Inter-expert kappa	0.408 (95% CI 0.254–0.562), moderate	Gap of nearly 18 percentage points in sensitivity among three radiologists (Ghafoor)	Consistent with prior variability; first formal report using Cohen's kappa in a female cohort
Reclassification due to extraperitoneal fat	1.0% (5/494) of the total cohort	Coexisting cord lipoma in a substantial fraction of laparoscopic repairs; proposed to be treated as a functional hernia (14, 15)	Novel contribution: first quantitative report in a female cohort evaluated by CT in the region

The 2-of-3 majority rule requires no external arbiters or in-person consensus. It resolves discordance with an explicit, prespecified, and case-traceable criterion. Combined with transparent reporting of the three readings and the adjudicated verdict, it preserves auditability and satisfies STROBE recommendations on transparency (16). In a Latin American context where diagnostic studies with more than two readers are infrequent, this architecture offers a practical alternative that does not depend on additional economic resources but on prior operational organization.

Strengths include an exclusively female cohort—unusual in imaging-based hernia literature—directly addressing the 84% male bias flagged by Piga et al. (12). The cohort was not selected by hernia suspicion, so the estimated frequency reflects real radiologic prevalence in women consulting for diverse indications. Executed size (494) was 2.7 times the planned 184, increasing power without compromising objectives. Dual expert reading with 2-of-3 adjudication allows formal quantification of interobserver variability. Limitations include the retrospective single-center design, 18 pending Reviewer 2 rereadings at closure, lack of systematic surgical verification and absence of a surgical reference standard for absolute sensitivity/specificity, absence of Valsalva maneuver in the original acquisition protocol (reducing sensitivity for small indirect inguinal hernias) (23, 26), and free-text reporting of defect size limiting numerical quantification in a fraction of cases.

## Conclusions

In this Colombian female cohort, the adjudicated prevalence of inguinal and femoral hernias by abdominal CT reached 16.8% (95% CI 13.8–20.4), substantially above the classical clinical range of 3%–6% for women (5). Extraperitoneal fat protrusion was documented as a differential diagnosis in 1.0% of studies, concentrating a large share of resident–expert discordance. Interobserver concordance ranged from almost perfect to fair depending on the expert reviewer, and inter-expert concordance was moderate (22). Incidental CT detection in non-hernia-specific examinations represents a high-yield diagnostic window in adult women consulting for other causes.

This work provides methodological and epidemiological contributions. Methodologically, systematic characterization of the extraperitoneal fat reclassification pattern in a female cohort is a poorly described element in regional literature; the 2-of-3 majority rule offers a transferable architecture for diagnostic studies with limited resources. Epidemiologically, the exclusively female, non-suspicion-selected cohort partially compensates for the 84% male bias flagged by Piga et al. (12). The 16.8% figure provides a quantitative referent for future discussions on incidental hernia reporting in Colombian reference institutions.

Three practical recommendations derive from the findings. First, systematically incorporate the diagnostic pair “true inguinal hernia vs. extraperitoneal fat protrusion” in tomographic reporting, with explicit anatomic criteria that orient the requesting clinician and reduce prevalence overestimation. Second, standardize incidental reporting of hernias identified on CT performed for other causes, with uniform language on laterality, subtype, sac content, and complications (8). Third, where volume and resources permit, incorporate double-reading or second-reviewer adjudication structures for subtle incidental diagnosis; the 2-of-3 rule documented here requires neither external arbiters nor in-person consensus. Future research should include multicenter prospective cohorts across Colombian regions, surgical validation for absolute sensitivity and specificity, dedicated studies on extraperitoneal fat protrusion in women with specific imaging protocols and anatomic validation, and cost-effectiveness analyses of standardized incidental reporting.

## Data Availability

The raw data supporting the conclusions of this article will be made available by the authors without undue reservation.
